# NF-κB-dependent Mechanism of Action of c-Myc Inhibitor 10058-F4: Highlighting a Promising Effect of c-Myc Inhibition in Leukemia Cells, Irrespective of p53 Status

**DOI:** 10.22037/ijpr.2020.112926.14018

**Published:** 2020

**Authors:** Mohammad Sayyadi, Ava Safaroghli-Azar, Majid Safa, Hassan Abolghasemi, Majid Momeny, Davood Bashash

**Affiliations:** a *Department of Hematology and Blood Banking, School of Allied Medical Sciences, Shahid Beheshti University of Medical Sciences, Tehran, Iran. *; b *Department of Hematology and Blood Banking, School of Allied Medical Sciences, Iran University of Medical Sciences, Tehran, Iran. *; c *Pediatric Congenital Hematologic Disorders Research Center, Shahid Beheshti University of Medical Sciences, Tehran, Iran. *; d *Cancer Cell Signaling, Turku Center for Biotechnology, University of Turku and * *Åbo* * Akademi University, Turku, Finland.*

**Keywords:** 10058-F4, c-Myc, p53, NF-κB pathway, PI3K pathway, Autophagy

## Abstract

Due to the frequent contribution in the pathogenesis of different human malignancies, c-Myc is among those transcription factors that are believed to be pharmacologically targeted for cancer therapeutic approaches. In the present study, we examined the anti-leukemic effect of a well-known c-Myc inhibitor 10058-F4 on a panel of hematologic malignant cells harboring either mutant or wild-type p53. Notably, we found that the suppression of c-Myc was coupled with the reduction in the survival of all the tested leukemic cells; however, as far as we are aware, this study suggests for the first time that the cytotoxic effect of 10058-F4 was not significantly affected by the molecular status of p53. Delving into the molecular mechanisms of the inhibitor in the most sensitive cell line revealed that 10058-F4 could induce apoptotic cell death in mutant p53-expressing NB4 cells through the suppression of NF-κB pathway coupled with a significant induction of intracellular reactive oxygen species (ROS). In addition, we found that the anti-leukemic effect of 10058-F4 was overshadowed, at least partially, through the compensatory activation of the PI3K signaling pathway; highlighting a plausible attenuating role of this axis on 10058-F4 cytotoxicity. In conclusion, the results of the present study shed light on the favorable anti-leukemic effect of 10058-F4, especially in combination with PI3K inhibitors in acute promyelocytic leukemia; however, further investigations should be accomplished to determine the efficacy of the inhibitor, either as a single agent or in a combined-modal strategy, in leukemia treatment.

## Introduction

Upon the first description of MYC gene in the human genome, it was believed that this protein only serves as a transcription factor (1, 2). However, as the biological characteristics became clearer, it has been suggested that c-Myc plays a key role in the regulation of more biological disciplines, including cell proliferation, cell metabolism, and cell survival (3). As its role in tumor progression emerges, numerous studies have peered into the therapeutic ability of this onco-protein, either as a prognostic biomarker or as an appealing target for cancer treatment. In a study conducted by Nalan *et al.*, it was reported that rearrangement of c-Myc in diffuse large B cell lymphoma (DLBCL) could be an effective biomarker to predict high risk patients (4). In another study, it has also been indicated that over-expression of c-Myc could alter the expression level of apoptosis-related genes, playing a key role in the development of mantle cell lymphoma (5). The association between c-Myc overexpression and the incidence of human hematologic malignancies, in particular acute leukemia, has also been well-established in numerous studies (6, 7). Being the most deregulated proto-oncogene in hematologic malignances, it has been reported that aberrant c-Myc expression is frequently associated with poor prognosis (8).

Owing to the aberrant expression of c-Myc, numerous attempts have been launched to identify pharmacologic inhibitors of c-Myc for therapeutic applications. Computer-aided drug discoveries have lately laid a foundation for developing potent and clinically optimized c-Myc inhibitors that could serve as favorable therapeutics (9). Given to its favorable anti-cancer property, 10058-F4 is among those c-Myc inhibitors that are believed to bring advantages for the treatment of various types of cancers, especially in the term of combination therapy. The remarkable anti-cancer property of this inhibitor has been reported in different solid tumors including ovarian cancer (10), hepatocellular carcinoma (11), prostate cancer (9), and hypopharyngeal carcinoma (12), as well as hematologic malignancies (13, 14). In a more recent investigations, it was also delineated that co-targeting c-Myc and other components of signaling pathways, for instance PI3K (15), PAK (16), and ERK (17) may be more efficient in reducing the number of cancer cells in human malignancies. Moreover, it has been suggested that the suppression of c-Myc could be a promising strategy to enhance the anti-leukemic property of vincristine, a potent chemotherapeutic drug used in the first line treatment of acute lymphoblastic leukemia (14, 18). Although the anti-tumor effects of the inhibitor has turned into an area of focus in the recent years, the underlying molecular mechanisms through which 10058-F4 exerts its favorable effects has not yet been described properly. To the best of our knowledge, to date, no study has addressed the potential role of p53 status in leukemic cell response to 10058-F4 and this study suggested for the first time that the favorable cytotoxic effect of 10058-F4 in leukemic cells could not be swayed by the molecular status of p53. We found that the superior cytotoxic effect of this agent in 53-mutant expressing NB4 cells, as the most sensitive cell line, is mediated probably through NF-κB-mediated augmentation reactive oxygen species (ROS) level.

## Experimental

*Cell culture and drug treatment*

To investigate the effects of 10058-F4 on hematologic malignant cell lines, a panel of cells consists of U937, Nalm-6, REH, KG-1, KMM-1, HL-60, and NB4 (Pasteur Institute, Tehran, Iran) cells were grown in RPMI 1640 medium supplemented with antibiotics, 10% fetal bovine serum (Invitrogen) and 2 mM l-glutamine (Invitrogen) in the presence of 5% CO_2_ at 37 °C. The relevant amount of 10058-F4 was dissolved in sterile PBS to make a stock solution. Moreover, PI3K inhibitor CAL-101 (Selleckchem, Munich, Germany), proteasome inhibitor Carfilzomib (Selleckchem, Munich, Germany), and autophagy inhibitor chloroquine (CQ) (Sigma, Taufkirchen, Germany) were prepared, divided to aliquots, and stored at −20 °C until use.

*Trypan blue assay test of cell count and viability*

To assess the suppressive effect of 10058-F4 on the viability and growth kinetics, hematologic malignant cell lines were incubated with designated concentrations of c-Myc inhibitor, either alone or in combination with several anti-cancer agents. After indicated treatment intervals, the drug-treated cells were mixed with 0.4% trypan blue solution (Invitrogen) in a 1:1 ratio and then the mixture was allowed to incubate for 1–2 min at room temperature and loaded onto the chamber of Neubauer hemocytometer. The total number of unstained (viable) and the stained (non-viable) cells were manually counted and the percentage of viable cells was calculated.

*Detection of metabolic activity by micro-culture tetrazolium test*

To assess whether the treatment of the cells 10058-F4, as a single agent or in combined modality, could reduce the metabolic activity, the hematologic malignant cells were treated with different concentrations of this c-Myc inhibitor, either alone or in combination with other anti-leukemic agents. After treatment of the cells with different agents, we incubated the cells with 100 μL of MTT solution for a further 3 h in a humidified incubator. The optical densitometry of a resulting formazan solubilized with DMSO was measured in an enzyme-linked immunosorbent assay (ELISA) reader at the wavelength of 570 nm. The percentage of metabolic activity was calculated as (%) = (OD_exp_/OD_con_) × 100; where OD_exp_ and OD_con_ are the optical densities of exposed and control cells, respectively.

*BrdU cell proliferation assay*

The suppressive effect of 10058-F4 on the growth and proliferation of NB4 cells was assessed by measuring DNA synthesis rate of the cells using a colorimetric bromodeoxyuridine (BrdU)-based cell proliferation ELISA kit (Roche, Penzberg, Germany) as per manufacturer’s recommendations. Initially, APL-derived cells were seeded into 96-well plate at a density of 5000 cells/well and treated with the desired concentrations of 10058-F4. Afterwards, 10 μL/well of BrdU labeling solution was added, and the cells were re-incubated at 37 °C for 12 h. Then, the cells were then fixed and DNA was denatured using 200 μL of FixDenat solution provided with the kit. After 30 min and discarding Fixodent, 100 μL peroxidase-conjugated anti-BrdU (antiBrdU-POD) antibody was added to each well. Finally, the cells were incubated with tetramethylbenzidine (TMB) for 3 min at room temperature and the reaction product was quantified by measuring the absorbance at 450 nm.

*Cell cycle distribution analysis*

The impact of 10058-F4 on the distribution of the cells in the different phases of the cell cycle were ascertained by ﬂow cytometric analysis after 24 h incubation of NB4 cells with different concentrations of the inhibitor. In brief, 1 × 10^6^ cells were harvested, washed twice with cold PBS, and then ﬁxed in 70% ethanol. Next, propidium iodide (PI) and RNase were used to stain DNA and degrade RNA, respectively. DNA content of the cells was quantified from the peak analysis of flowcytometric histograms, and the data were interpreted using the Windows FlowJo™ v10 software.

*Assessment of apoptosis using ﬂow cytometry*

The apoptotic effect of 10058-F4, either alone or in combination with CAL-101 against APL-derived cells were investigated by Annexin-PI staining assay. Briefly, the cells were harvested after 24 h of treatment with the designated concentrations of the inhibitor, washed with PBS, and re-suspended in a total volume of 100 μL of the incubation buﬀer at a concentration of 1 × 10^6^ cells/mL. After that, Annexin-V-Flous (2 μL per sample) was added, and the cell suspensions were incubated for 20 min in the dark. After incubation, Fluorescence was quantified using flow cytometry.

*Measurement of caspase-3 enzymatic activity*

To determine whether 10058-F4-induced apoptosis is mediated through caspase-dependent cascade, we investigated the enzymatic activity of caspase-3 using a caspase-3 assay kit (Sigma). Briefly, the cells were treated with 100, 150, and 200 μM 10058-F4 and incubated at 37 °C for 24 h. Following centrifugation at 600 ×g for 5 min, the cell pellets were lysed and the lysates were centrifuged at 20,000 ×g for 10 min. In a total volume of 100 μL, 5 μg of the supernatant was incubated with 85 μL of assay buffer plus 10 μL of caspase-3 substrate in a 96-well plate at 37 °C. The cleavage of the peptide by caspase-3 released the chromophore pNA, which was quantified spectrophotometrically at 405 nm.

*Western blot analysis*

NB4 cells were centrifuged after treatment with 100 and 150 µM of 10058-F4, and the cellular pellets were lysed in RIPA buffer containing protease and phosphatase inhibitor cocktails (Sigma). The protein concentration was determined according to Bradford method and then equivalent amounts of total cellular protein were separated by 10% SDS-PAGE, and subsequently transferred to nitrocellulose membrane using a semidry transfer cell (Bio-Rad). Afterwards, the membranes were blocked with 5% non-fat dry milk in TBS containing 0.1% (v/v) Tween-20 for 1 h at room temperature. The proteins were detected using specific primary antibodies against IκB, p- IκB, Akt, p-Akt, cleaved PARP, β-actin, cleaved PARP (cell signaling), and cleaved caspase-3 (Abcam), and the enhanced chemiluminescence detection system according to the manufacturers protocol.

*RNA extraction and cDNA synthesis*

Total RNA from 10058-F4-treated cells was extracted using RNA Isolation Kit (Roche, Mannheim, Germany) and quantified by Nanodrop instrument. The reverse transcription reaction was performed using Complementary DNA (cDNA) Synthesis Kit (Takara Bio, Shiga, Japan). Adapted times and temperature profiles for the reverse transcription were used: incubation for 5 min at 65 °C, 5 min at 25 °C, followed by 60 min at 42 °C. The reaction was terminated by heating for 5 min at 70 °C.

*Quantitative real-time PCR*

Changes in mRNA expression level of the desired genes were assessed by real-time PCR that was performed with a light cycler instrument (Roche Diagnostics, Germany) using SYBR Premix Ex Taq technology (Takara Bio, Inc.). For this purpose, PCR assay was performed in an ultimate volume of 20 μL of reaction mixture containing 10 μL of SYBR Green master mix, 2 μL of cDNA product, 0.5 μL of each forward and reverse primers (10 pmol) and 7 μL of nuclease-free water (Qiagen, Hilden, Germany). Thermal cycling conditions included an initial activation step for 30 s at 95 °C followed by 40 cycles including a denaturation step for 5s at 95 °C and a combined annealing/extension step for 20 s at 60 °C. Melting curves were analyzed to verify single PCR product of each primer ABL housekeeping gene amplified as an internal control, and fold change in the expression of each target mRNA relative to ABL was calculated on the basis of a comparative on 2^-ΔΔct^ relative expression formula.

*Intracellular reactive oxygen species detection*

To determine the effect of 10058-F4 on the amount of intracellular reactive oxygen species (ROS) in NB4 cells, we used a fluorogenic dye DCFH-DA, for measuring hydroxyl, peroxyl and other ROS activity within the cell. After incubation with the desired concentrations of the nanocomposite, the cells were incubated with DCFH-DA at 37 °C for 30 min. Finally, fluorescence intensities of the samples were detected by fluorescence spectrophotometer (Cary Eclipse, USA) with excitation at 485 nm and emission at 530 nm.

*Statistical analysis*

Experimental data were evaluated in triplicate against untreated control cells and collected from three independent experiments. The signiﬁcance of differences between experimental variables was determined by the use of two tailed student’s test. In order to compare between the control group and the treated ones, the Dunnett’s multiple comparison test was used. All data are presented as mean ± standard deviation (SD) and a probability level of *P* ≤ 0.05 was considered statistically significant.

## Results

*Abrogation of c-Myc using 10058-F4 reduced survival of hematologic malignant cell lines*

**Figure 1:**
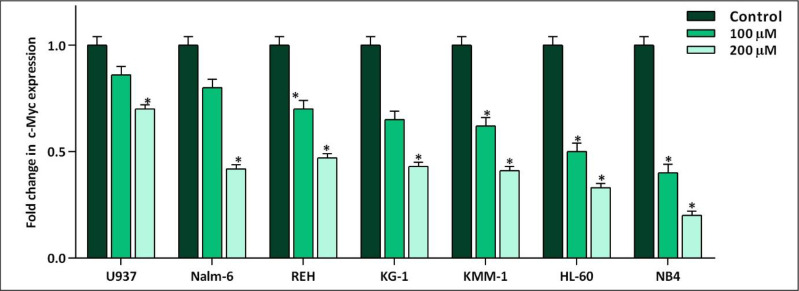
The effect of 10058-F4 on the expression level of c-Myc. After exposing the panel of hematologic malignant cell lines with 10058-F4 at the concentrations of 100 and 200 µM, the mRNA expression level of c-Myc was evaluated. Values are given as mean ± standard deviation of three independent experiments. ^*^P ≤ 0.05 represented significant changes from the control

**Figure 2:**
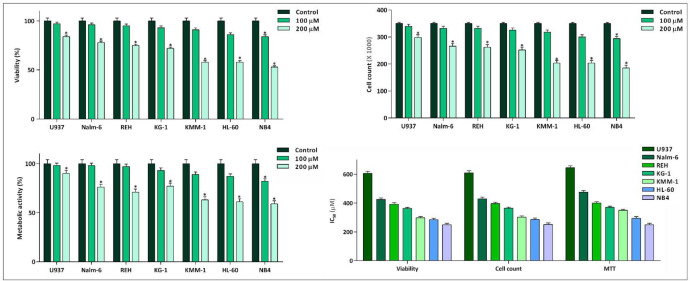
Inhibitory effect of 10058-F4 on viability, cell count, and metabolic activity of leukemic cell lines. 10058-F4 induced anti-leukemic effect in all cell lines; however, a different cell sensitivity pattern was noted among the tested cells. Values are given as mean ± standard deviation of three independent experiments. ^*^P ≤ 0.05 represented significant changes from the control

**Figure 3:**
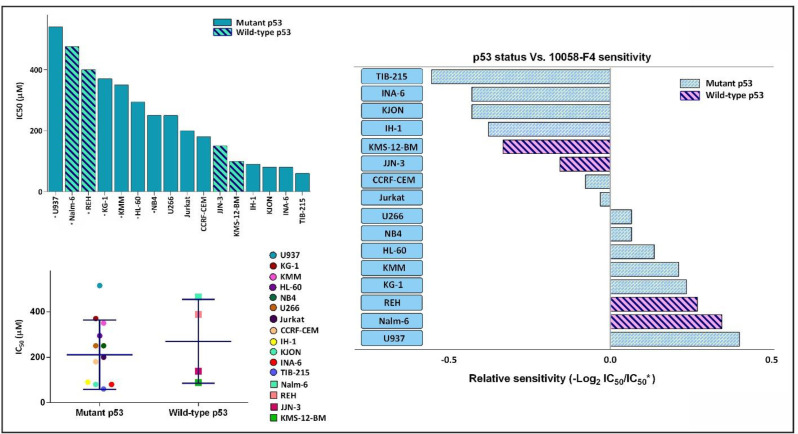
The anti-leukemic effect of 10058-F4 on hematologic malignant cell lines is exerted irrespective of the molecular status of p53. Based on our supplemental investigation and an extensive literature review, a list of IC_50_ response of different leukemic cell lines to 10058-F4 after 24 h was made. IC_50_ of starred cell lines was evaluated in our laboratory. Dot blot showing correlation between p53 status and *in-vitro* drug sensitivity as shown by the IC_50_ of individual cell line. Lines indicate median value. We failed to identify an obvious association between p53 status and leukemic cell sensitivity to 10058-F4. Values are given as mean ± standard deviation of three independent experiments

Not so long time after the discovery of the fundamental role of c-Myc in immortalization of malignant cells in different human cancers, the specific small molecule inhibitors of this factor have found their way in the route of targeted therapies (19). Our results in a panel of leukemic cells showed that not only 10058-F4 could decrease c-Myc expression level (Figure 1). We also found that c-Myc suppression using 10058-F4 potently decreased viability, cell count and metabolic activity of all the cell lines; however, the IC_50_ values varied among the tested cells (Figure 2). Ample genetic and laboratory studies suggest that the promoter of c-Myc has a binding site for p53 tumor suppressor protein (20). Given this, it was attractive to examine whether the difference in the sensitivity pattern of the cells to 10058-F4 could be as a results of the molecular status of p53. Based on our supplemental investigations on a panel of hematologic malignant cell lines, we failed to identify any significant linking between molecular status of p53 and cell response to 10058-F4 (Figure 3), indicating the potential application of the inhibitor either in wild-type or deficient p53-expressing leukemic cells.

*10058-F4 reduced NB4 cell viability through induction of a caspase-3-dependent apoptosis*

**Figure 4:**
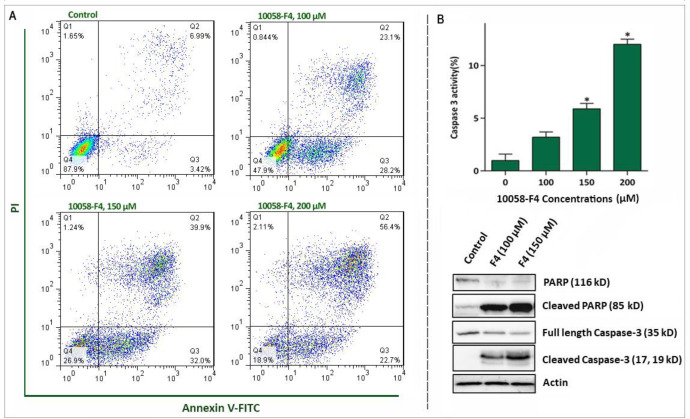
Abrogation of c-Myc induced caspase‐3‐dependent apoptosis in NB4 cell lines. (A) Treatment of NB4 cells with 10058-F4 remarkably increased the percentages of Annexin-V/PI positive cells. (B) 10058-F4 imposed a considerable elevation in caspase-3 activity and increased the amount of cleaved caspase 3 and PAPR. Values are given as mean ± SD of three independent experiments. ^*^P ≤ 0.05 represented significant changes from the control

To precisely delve into the underlying mechanisms responsible for the anti-leukemic effect of 10058-F4 on the most sensitive cell line, we chose APL-derived NB4 cells for further experiments. In agreement with the results of trypan blue and MTT assays, FACS analysis of Annexin-V/PI staining revealed that the treatment of NB4 cells with c-Myc inhibitor led to a considerable increase in Annexin-V/PI double positive cells (Figure 4A). Induction of apoptosis in NB4 was also authenticated by western blot and caspase-3 activity assays, which disclosed that the apoptotic cell death is primarily due to the induction of a caspase-3-mediated apoptosis in NB4 cells. As presented in Figure 4B, we found that 10058-F4 increased the amount of cleaved PARP and caspase-3 and up-regulated the enzymatic activity of this enzyme in a concentration-dependent manner.

*The anti-leukemic effect of 10058-F4 on NB4 cells is mediated through suppression of NF-* *κB* *pathway*

**Figure 5:**
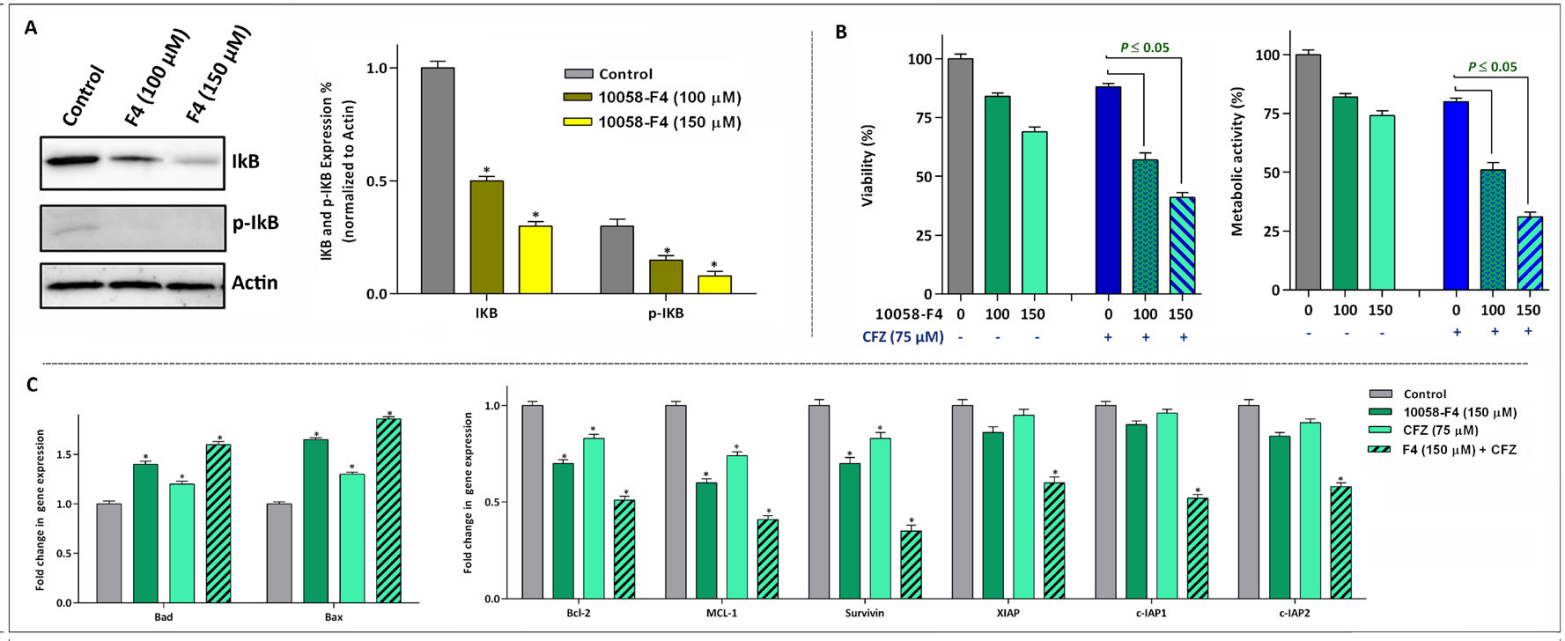
The anti-leukemic effect of 10058-F4 is mediated through suppression of NF-κB and its associated genes. (A) After the treatment of cells with the indicated concentrations of 10058-F4 for 24 h, total cell lysates were prepared and western blotting was performed using antibodies specific to IκB, p-IκB and Actin. (B) Suppression of proteasome using carfilzomib (CFZ) potentiate the anti-leukemic effect of 10058-F4. (C) The results of qRT-PCR analysis revealed that expression of both pro- and anti-apoptotic target genes altered more significantly when 10058-F4 was used in combination with CFZ. Values are given as mean ± SD of three independent experiments. ^*^P ≤ 0.05 represented significant changes from the control

From the first description of c-Myc in leukemogenesis, its tight association with the NF-κB signaling pathway has been investigated in several reports (21), hinting this point that the anti-leukemic effect of c-Myc inhibition may be mediated through modulation of NF-κB. Of note, the results of western blot analysis revealed that 10058-F4 potently reduced both the expression and phosphorylation level of IκB in NB4 cells (Figure 5A). Accordingly, we found that the suppression of NF-κB axis using a well-known proteasome inhibitor carfilzomib (CFZ) enhanced the ability of 10058-F4 to reduce survival and proliferation capacity of NB4; shedding more light on the contributory role of NF-κB pathway on the mechanism of action of 10058-F4 (Figure 5B). The results of qRT-PCR analysis also revealed that combination of 10058-F4 with carfilzomib remarkably reduced the survival of NB4 cells through alteration in the expression level of both anti- and pro-apoptotic-related genes (Figure 5C).

*10058-F4 impeded proliferation of NB4 through ROS-mediated alteration of cell cycle*

**Figure 6:**
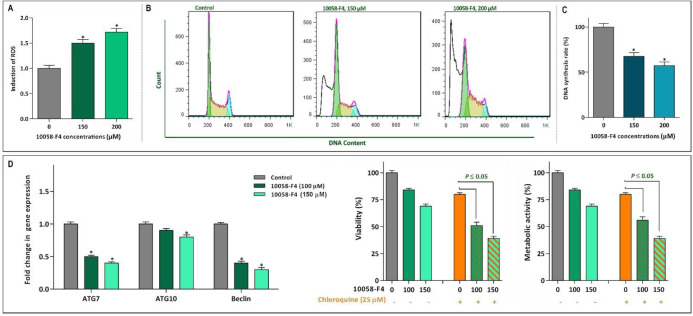
Effect of 10058-F4 on ROS level, the cell cycle progression and autophagy system. (A) Upon treatment of NB4 cells with 1008-F4, the intercellular level of ROS was elevated in a concentrations-dependent manner. (B) Treatment of the cells with 10058-F4 increased the proportion of NB4 cells in sub-G1 phase, while there was a reduction in the percentage of cells S phase. (C) The results of BrdU assay showed that 10058‐F4 could hamper the replicative potential of APL cells through reducing DNA synthesis rate. (D) The single agent of 10058-F4 could remarkably diminished the expression level of autophagy-related genes in NB4 cells. Moreover, when autophagy system was suppressed using chloroquine, the cytotoxic effect of 10058-F4 was remarkably reinforced. Values are given as mean ± SD of three independent experiments. ^*^P ≤ 0.05 represented significant changes from the control

Based on the suppressive effect of 10058-F4 on NF-κB signaling pathway and given to the penetrance of this axis on the generation of intracellular reactive oxygen species (ROS) (22), we aimed to examine whether there was a correlation between c-Myc inhibition and ROS production in NB4. Our results showed that 10058-F4 remarkably augmented the intracellular level of ROS in inhibitor-treated cells in a concentration-dependent manner (Figure 6A). It has been reported that activation of ROS in cancer cells could impaire the replication of DNA in S phase of cell cycle making malignant cells more vulnerable to the death stimuli (23). In agreement with the elevation of ROS, DNA content analysis revealed that 10058-F4 potently reduced the percentage of NB4 cells in S phase of cell cycle (Figure 6B). This finding was further straighten out with BrdU incorporation assay indicating that there is a concentration-dependent reduction in the DNA synthesis rate of NB4 cells after exposure to 10058-F4 (Figure 6C). Moreover, cell cycle analysis by flow cytometry revealed that the percentage of hypodiploid cells, which are detected by so-called sub-G1 peak, significantly elevated which is in agreement with the results of Annexin-PI staining assay.

*The attenuating effect of autophagy on the anti-leukemic effect of 10058-F4 in NB4 cells*

**Figure 7.**
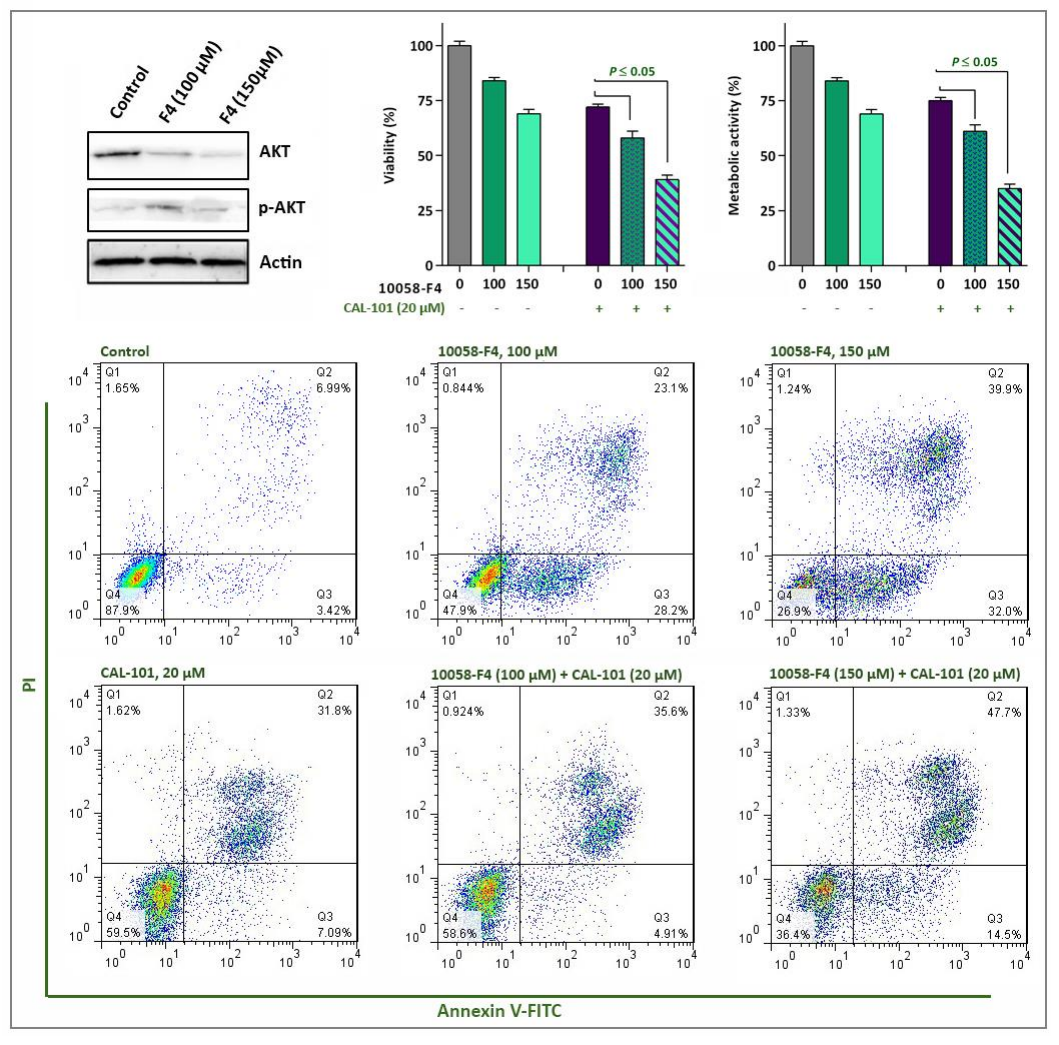
The effect of 10058-F4 on the PI3K signaling pathway. The results of western blot analyzing revealed that upon c-Myc inhibition the amount of phosphorylated Akt increased in NB4 cells. When 10058-F4 was accompanied by a PI3K inhibitor, CAL-101, the survival of NB4 cells were decreased more efficiently as compared to either agents alone. Values are given as mean ± SD of three independent experiments. ^*^P ≤ 0.05 represented significant changes from the control

Acting in contrast to ROS, activation of autophagy system in malignant cells has been reported to aid tumor cells to survive for the longer period of time (24). We found that 10058-F4 potently suppressed the induction of autophagy through down-regulation of genes involved in the formation of autophagosome (Figure 6D). Moreover, our results showed that when autophagy system was inhibited in NB4 cells using the non-toxic concentration of chloroquine, the anti-leukemic property of 10058-F4 was enhanced more significantly (Figure 6D), suggestive of the attenuating role of autophagy on the cytotoxicity of the inhibitor. Playing in a triangle, the tight correlation between the autophagy system, the PI3K signaling pathway, and c-Myc has been investigated in several studies (25, 26). Noteworthy, when we examined the effect of 10058-F4 on the PI3K signaling pathway, we found that the amount of phosphorylated Akt, as the most critical component of the PI3K pathway, was elevated in response to the inhibitor which could be probably due to the malignant cells desire for longer survival (Figure 7). In agreement, co-treatment with PI3K inhibitor CAL-101 and c-Myc inhibitor 10058-F4 remarkably diminished the survival capacity of NB4 cells (Figure 7), which may explain, at least partially, the compensatory role of the PI3K pathway upon c-Myc suppression. This finding was then substantiated by the results of Annexin-V staining assay which revealed that the proportion of apoptotic cells elevated dramatically when NB4 cells were exposed to both c-Myc and PI3K inhibitors (Figure 7).

**Figure 8:**
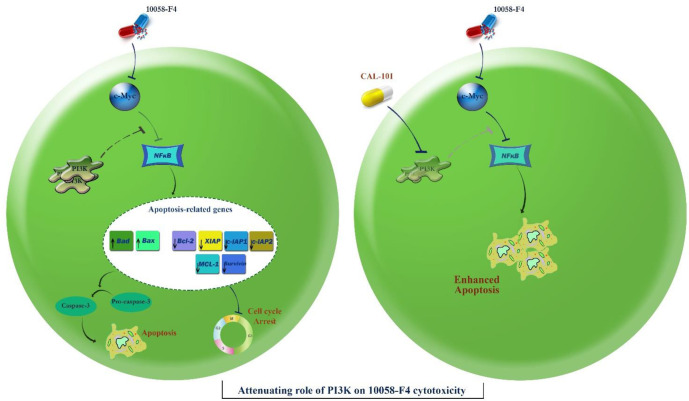
Schematic representation proposed for the plausible mechanisms of action of 10058-F4 in APL-derived NB4 cells. Through inhibition of c-Myc, 10058-F4 augmented the intracellular level of ROS and induced caspase-3-dependent apoptotic cell death in NB4 cells through suppression of the NF-κB signaling pathway. This favorable anti-leukemic activity could be attenuated through activation of the PI3K signaling pathway. Suppression of the PI3K axis using CAL-101 eliminated the compensatory effect of this pathway on 10058-F4-induced cytotoxic effect and promoted a more significant apoptotic cell death in NB4 cells

## Discussion

Soon after the discovery of MYC family genes and their involvement in oncogenic processes spanning from the regulation of cell growth to the maintenance of cancer cell survival, it was postulated that c-Myc inhibition could be translated into therapeutic approaches (8). The results obtained from the present study outlined that abrogation of c-Myc using novel specific inhibitor 10058-F4 remarkably reduced the survival and proliferative capacity of a panel of acute leukemia cell lines harboring an overexpressed c-Myc; however, a different cell sensitivity pattern was noted in response to the inhibitor. Given to the fact that c-Myc has a binding site on its promotor for p53 and its expression could be affected by the activity of this tumor suppressor (20), it was reasonable to hypothesize that the molecular status of p53 may influence on the extent of cell response to 10058-F4. Notably, our supplementary experiments revealed that there was no significant correlation between molecular status of p53 and leukemic cell response, which was in agreement with a previous disclosure which also failed to find any differential sensitivity pattern with respect to PTEN status (13). Taken together, it is assumed that the anti-leukemic effect of 10058-F4 is probably mediated regardless of the molecular status of PTEN and p53; highlighting the efficacy of this inhibitor in either wild-type or mutan PTEN and/or p53 leukemic cells.

The favorable anti-leukemic effect of the inhibitor on the most sensitive cell line was substantiated by apoptosis analysis, where we found that the lower concentrations of 10058-F4 induced a caspase-3-dependent apoptosis in APL-derived NB4 cells. It has been reported that active metabolism and genetic instability under the control of oncogenic transformation such as over-activation of c-Myc could provide a platform for cancer cells to harbor an excess oxidative stress level (27). Moreover, previous studies delineated that treatment of mutant p53-expressing leukemic cells with different small molecule inhibitors could increase the amount of ROS level (28, 29), proposing that probably the absence of anti-oxidant regulatory protein p53 makes cells vulnerable to the cytotoxic effects of anti-cancer agents (30). Accordingly, our results clearly showed that abrogation of c-Myc in NB4 cells harboring mutant p53 not only increased the amount of ROS, but also increased the expression levels of Bad and Bax, as two potent apoptotic genes with a unique cross-talk with ROS. Hitherto, roles deviating from canonical activity of ROS have been demonstrated in a fair number of studies. It was reported that ROS production could provide a signal that in partnership with the extinguished NF-κB axis regulate cell cycle progression (21). Interestingly, we found that the decreased replication of DNA as well as reduced cell population in S phase was coupled with the suppression of NF-κB axis upon NB4 cells treatment with the inhibitor. Noteworthy, the inhibitory impact of 10058-F4 on NF-κB network became more prominent where we found that the suppression of NF-κB using carfilzomib exerted a superior cytotoxic effects in NB4.

Acting as a double edged sword, induction of autophagy could either trigger cell death or induce a resistance phenotype, according to the cancer cell type (13, 14 and 31). Consistently, investigating the effects of 10058-F4, which as a single agent reduced the expression levels of autophagy-related genes, in combination with a well-known autophagy inhibitor revealed that the inhibition of autophagy not only resulted in a concentration-dependent cytotoxicity, but also exerted a superior cytotoxicity in inhibitors-treated NB4 cells; indicating that the anti-leukemic effect of 10058-F4 was mediated, at least partly, through the suppression of autophagy. Playing in a triangle, the tight correlation between the autophagy system, the PI3K signaling pathway and c-Myc has been investigated in several studies. In a study conducted by Balakumaran *et al.*, it has been suggested that activation of c-Myc in prostate cancer cells could on one hand activate the PI3K signaling pathway, and on the other hand regulated the activity of autophagy system (26). By investigating the effect of 10058-F4 on the PI3K pathway, we found that the amount of phosphorylated Akt is elevated in response to the inhibitor which could be probably due to the great desire of malignant cells for longer survival. Notably, our results revealed that simultaneous blockade of PI3K and c-Myc remarkably diminished the survival capacity of NB4 cells, which may explain, at least partially, the compensatory role of the PI3K pathway upon 10058-F4 treatment (Figure 8). Taken together, the present study showed that 10058-F4 had a significant anti-tumor activity against hematologic malignant cells, especially in APL-derived cells. In addition, we reported for the first time that the anti-leukemic property of the inhibitor was exerted irrespective to the molecular status of p53; suggestive of the probable application of 10058-F4 in both mutant and wild-type p53-expressing leukemic cells.
